# Characterization of the First W-Specific Protein-Coding Gene for Sex Identification in *Helicoverpa armigera*

**DOI:** 10.3389/fgene.2020.00649

**Published:** 2020-06-19

**Authors:** Zhongyuan Deng, Yakun Zhang, Min Zhang, Jinyong Huang, Changyou Li, Xinzhi Ni, Xianchun Li

**Affiliations:** ^1^School of Agricultural Sciences, Zhengzhou University, Zhengzhou, China; ^2^State Key Laboratory for Biology of Plant Diseases and Insect Pests, Institute of Plant Protection, Chinese Academy of Agricultural Sciences, Beijing, China; ^3^College of Plant Health and Medicine, Qingdao Agricultural University, Qingdao, China; ^4^Agricultural Research Service, U.S. Department of Agriculture, Crop Genetics and Breeding Research Unit, University of Georgia – Tifton Campus, Tifton, GA, United States; ^5^Department of Entomology and BIO5 Institute, University of Arizona, Tucson, AZ, United States

**Keywords:** *doublesex*, egg transcriptome, sex identification, male genome, sexual dimorphism, transposon

## Abstract

*Helicoverpa armigera* is a globally-important crop pest with a WZ (female)/ZZ (male) sex chromosome system. The absence of discernible sexual dimorphism in its egg and larval stages makes it impossible to address any sex-related theoretical and applied questions before pupation unless a W-specific sequence marker is available for sex diagnosis. To this end, we used one pair of morphologically pre-sexed pupae to PCR-screen 17 non-transposon transcripts selected from 4855 W-linked candidate reads identified by mapping a publicly available egg transcriptome of both sexes to the male genome of this species and detected the read SRR1015458.67499 only in the female pupa. Subsequent PCR screenings of this read and the previously reported female-specific RAPD (random amplified polymorphic DNA) marker *AF18* with ten more pairs of pre-sexed pupae and different annealing positions and/or temperatures as well as its co-occurrence with the female-specific transcript splicing isoforms of *doublesex* gene of *H. armigera (Hadsx)* and amplification and sequencing of their 5′ unknown flanking sequences in three additional pairs of pre-sexed pupae verified that SRR1015458.67499 is a single copy protein-coding gene unique to W chromosome (named *GUW1*) while *AF18* is a multicopy MITE transposon located on various chromosomes. Test application of *GUW1* as a marker to sex 30 neonates of *H. armigera* yielded a female/male ratio of 1.14: 1.00. Both *GUW1* and *Hadsx* splicing isoforms assays revealed that the *H. armigera* embryo cell line QB-Ha-E-1 is a male cell line. Taken together, *GUW1* is not only a reliable DNA marker for sexing all stages of *H. armigera* and its cell lines, but also represents the first W-specific protein-coding gene in lepidopterans.

## Introduction

Sex has profound effects on almost all aspects of animal life. This is manifested by the ubiquity of sexually-dimorphic morphological, physiological, behavioral, and life-history traits (Allen et al., 2011; Mowrey and Portman, 2012). Most notably, males and females differ in a broad variety of morphological traits that are involved in feeding, mate location, dispersal, escape from natural enemies, and/or oviposition. These include wing size and shape, size and color of pigment patches (female- or male-limited mimicry) and UV-reflective regions on the wings, size ratio of body parts (e.g., thorax to abdomen size ratio), color and density of body hairs, size and shape of sensory structures (e.g., antennae, eyes, auditory organs), size and shape of genitalia, and pheromone-releasing structures (Allen et al., 2011). For night-flying insects, female is usually the signaling sex which releases sex pheromone at night to attract and call the signal-receiving male which has more elaborate antennae. For day-flying species, male is the signaling sex which displays its vivid UV-reflective wings to attract the visual signal-receiving females for mating (Allen et al., 2011). In general, males are often smaller than females in body size but emerge earlier than the latter (male protandry) (Fischer and Fiedler, 2001; Esperk et al., 2007; Stillwell et al., 2010; Teder, 2014). The sexual size dimorphism is resulted from sex differnce in devlopemt time or even number of larval instar (larger sex has a longer development time or even more number of larval instar) (Esperk et al., 2007; Teder, 2014). In most insects, larger females also have a longer lifespan than smaller males (Fox et al., 2003). Less universal dimorphic traits include higher tolerance to insecticides (Li et al., 1992), heat stress (Gruntenko et al., 2016), and pathogen infection (Vincent and Sharp, 2014) as well as lower tendency to enter diapuse and shorter post-diapause period (Shimizu and Fujisaki, 2002) in females. Some of these secondary sexually-dimorphic traits, such as larger females (greater fecundity) and male protandry (better chance to mate with females), are largely associated with different reproductive roles, whereas others have a mix of sexual and non-sexual functions [e.g., arctiid moth auditory apparatus (Weller et al., 1999)] or function exclusively in non-reproductive contexts [e.g., female-limited mimetic color patterns (Kunte, 2009; Stillwell et al., 2010; Allen et al., 2011)].

Although sex of an individual insect is determined genetically upon fertilization and developed early in embryogenesis (Salz, 2011; Bopp et al., 2014; Biedler and Tu, 2016), most of the aforementioned sexual differences are uncovered in adult and/or pupal stages. This is because most insects do not exhibit conspicuous sexual dimorphism before pupation, and thus their eggs and larvae are often morphologically indistinguishable. Yet, characterization of the upstream diverged primary signal, master binary switch gene and autoregulatory gene of the sex determination pathway in various insects necessitates sex identification of eggs and young larvae/nymphs. Having a reliable method to sex eggs and larvae/nymphs of insects is also required for many other theoretical and applied research, such as molecular mechanisms of sexual trait development (Prakash and Monteiro, 2016) and genetic control of insect pests (San Andres et al., 2007). While flow cytometry measurement of DNA content (Nakamura et al., 1990; Aron et al., 2003), microscopic observation of sex chromatin (Traut and Marec, 1996; Fuková et al., 2005), and quantitative PCR (qPCR) measurement of the copy number (two copies in males vs. one copy in females) of the conserved Z chromosome gene in Lepidoptera (Belousova et al., 2019) can be used to determine sex of morphologically indistinguishable stages of some species, PCR gel analysis of sex-specific DNA markers revealed by RAPD (random amplified polymorphic DNA; Abe et al., 1998; Niu et al., 2007) assays or sex-specific chromosome (Y or W)-unique DNA sequences identified by comparison of male vs. female genome sequences (Krzywinski et al., 2004; Carvalho and Clark, 2013; Hall et al., 2013; Koerich et al., 2016) offers a more straightforward and reliable tool for sex identification. Such PCR-based sexing method is available for *Ceratitis capitata* (Douglas et al., 2004; San Andres et al., 2007; Gabrieli et al., 2010), *Cydia pomonella* (Fuková et al., 2009), *Tribolium castaneum* (Lagisz et al., 2010), and *Gnatocerus cornutus* (Gotoh et al., 2016), but has yet to be developed in many other crop pests.

*Helicoverpa armigera* is one of the most destructive polyphagous pests with hundreds of host plants belong to over 40 plant families (Zalucki et al., 1994; Zhao et al., 1998). Like *Bombyx mori* and most of lepidopterans (Fujii and Shimada, 2007; Traut et al., 2007), *H. armigera* has a WZ sex determination system, in which males are homogametic sex (ZZ) and females are heterogametic sex (WZ; Zhao et al., 2005; Niu et al., 2007). Male and female adults of *H. armigera* can be reliably sexed by their external genitalia and wing markings (Siverly, 1947; Zhao et al., 2005). At the pupal stage, the relative distance between the reproductive hole and the excretion hole can be used to sex male and female pupae (Zhao et al., 2005). For larvae older than the 3rd instar, one may recognize male and female larvae by the dorsal line, valve line, plane, and/or male testes (Jiang and Hu, 1995; Chen et al., 2004). However, sexing larvae older then the 3rd instar not only takes significant amount of time, practice and expertise, but also has a high error rate. Moreover, there are no morphological characters that can differentiate male from female eggs and larvae younger than the 3rd instar. Therefore, it is necessary to develop a PCR-based sexing method using W chromosome-unique DNA sequence for identification of male and female eggs, larvae and even pupae.

So far, no W-linked sequences for *H. armigera* are presently characterized, and only one female-specific RAPD marker (named *AF18* hereafter) has been reported for this species (Niu et al., 2007). In this study, we first identified 4855 cDNA sequence reads putatively transcribed from the W chromosome of female eggs by mapping a publicly available egg transcriptome of both sexes (NCBI SRA dataset accession number SRP031603, Anantanawat, 2013) to the male genome of this species (Li et al., unpublished data). We then PCR-screened one pair of morphologically pre-sexed pupae for the presence of 17 non-transposon reads selected from the 4855 W-linked candidate sequences and detected the read SRR1015458.67499 in the female pupa, but not in the male pupa. We further PCR-screened ten more pairs of pre-sexed pupae with different annealing positions and/or temperatures (62 or 58°C) and found that varying the annealing position and/or temperature altered the female specificity of the previously reported RAPD marker *AF18*, but not the female specificity of SRR1015458.67499. We also used three additional pairs of pre-sexed pupae to check the co-occurrence of SRR1015458.67499 (one sequence read in SRP031603, Anantanawat, 2013) with the female-specific transcript splicing isoforms of *H. armigera doublesex* gene (Hadsx, Wang et al., 2014) and clone the 5′-flanking unknown sequences of SRR1015458.67499 and *AF18*. Finally, we tested the utility of SRR1015458.67499 as a marker to sex one embryo cell line and 30 neonate larvae of *H. armigera.* These experiments revealed that *AF18* is a MITE (miniature inverted-repeat transposable elements) transposon located in multiple loci on both W and other chromosomes, whereas SRR1015458.67499 represents a single copy protein-coding gene unique to W chromosome (called *GUW1*) and thus can be used as a reliable DNA marker for sexing all growth stages of *H. armigera*.

## Materials and Methods

### *H. armigera* Strain and Cell Line

The laboratory colony of *H. armigera* used in this study was established with about 1200 larvae collected in tobacco fields in Xuchang (Henan, China) in June 2016 The colony had been maintained in a growth chamber at 26 ± 0.5°C with a photoperiod of 16 h light: 8 h dark and a relative humidity of 75 ± 5% (for adults) or 50 ± 5% (for larvae). Larvae of the colony had been reared on wheat germ-containing artificial diets (Waldbauer et al., 1984), whereas the adult moths had been fed with 10% honey water.

We pre-sexed 14 pairs of pupae based on the relative distance between the excretion and reproduction holes of each pupa according to Zhao et al. (2005). The pre-sexed pupae and a “tester set” of 30 unsexed neonate larvae were flash-frozen in liquid nitrogen and stored at −80°C for subsequent DNA and RNA extraction.

The embryo cell line QB-Ha-E-1 of *H. armigera* sexed in this study was established from another population (Zheng et al., 2010). QB-Ha-E-1 was seeded at 5 × 10^5^ per mL in 15 mL flask and cultured at 28°C with Grace’s Insect Medium (Gibco/Life Technologies, New York, NY, United States) supplemented with 10% fetal bovine serum (Gibco/Life Technologies, New York, NY, United States), 50 U/mL penicillin and 50 μg/mL streptomycin (HyClone, Thermo Fisher Scientific, Logan, UT, United States). After three days, the cells were pelleted down by centrifuge at 300 × *g*, flash-frozen in liquid nitrogen and stored at −80°C for subsequent DNA and RNA extraction.

### DNA/RNA Extraction and cDNA Synthesis

Genomic DNA (gDNA) was extracted from each of the 30 unsexed neonates as described (Li et al., 2002). Each of the 14 pairs of sexed pupae was individually grounded to powder in liquid nitrogen. Half of the powder was used to extract gDNA (Li et al., 2002). The other half was used to extract total RNA with the Trizol Regent (Life Technologies, Grand Island, NY, United States) according to the manufacturer’s manual. Likewise, half of the QB-Ha-E-1 cell pellet was used to extract gDNA and the other half for extraction of total RNA. The obtained DNA pellet from each neonate, pupa or the QB-Ha-E-1 cells was dissolved in the double distilled water (ddH_2_O), quantified using a NanoDrop 1000 instrument (NanoDrop, Wilmington, DE, United States), and then stored at −20°C.

The total RNA sample obtained from each pupa or the QB-Ha-E-1 cells was treated with DNase I (Promega, Madison, WI, United States) and RNase inhibitor (Thermo Fisher Scientific, Wilmington, DE, United States) for 40 min to remove potential gDNA contamination, cleaned with phenol/chloroform extraction, and dissolved in diethyl pyrocarbonate (DEPC) treated-H_2_O. The concentration of each cleaned RNA sample was determined using a NanoDrop 1000 instrument. We transcribed 1 μg of each RNA sample into cDNA using a mixture of random hexamer primers and oligo(dT)_20_ and Quant Reverse transcriptase (Tiangen Biotech, Beijing, China).

### Genomic PCR and RT-PCR Gel Analyses

We designed a pair of primers for each of the 17 selected putative W-linked transcript 454 reads (see the corresponding primers in Supplementary Table S1) and the reference gene *elongation factor 1 alpha* (*EF-1*α*;* present in both males and females; Supplementary Table S2) with Primer Premier 5.0 (Premier Biosoft International, Palo Alto, CA, United States) for the initial genomic PCR (gPCR) verification of their W chromosome uniqueness. For SRR1015458.67499 (i.e., *GUW1*), the only verified W-specific transcript in the initial PCR screen, we designed another pair of primers (GUW1-118-F and GUW1-118-R in Supplementary Table S2) for further verification of its W chromosome uniqueness and utility as a female specific molecular marker, and one gene-specific reverse primer GUW1-118-R (Supplementary Table S2 and Figure 5A) for amplification of its 5′-flanking sequences by genome walking. For the previously-reported female-specific RAPD marker *AF18* (Niu et al., 2007), we used the same AF18-F and AF18-R primers (Supplementary Table S2) reported in Niu et al. (2007) for its initial verification. We also designed another pair of primers AF18-436-F3 and AF18-436-R3 for its further verification and another gene-specific reverse primer AF-18-GSP (see these primers in Supplementary Table S2) for amplification of its 5′-flanking sequences by genome walking. All the primers that we designed have a Tm value of 60°C and thus the related gPCR reactions for the 17 putative W-linked transcript 454 reads and *EF-1*α used the same PCR set up and cycling conditions (see below) except for the genome working PCRs and the PCR reactions of *AF18*. For RT-PCR detection of sex-specific transcript splicing isoforms of the *doublesex* gene, we used the primer pair Hadsx-F and Hadsx-R (Supplementary Table S2) and cycling conditions as reported in Wang et al. (2014).

The gPCR reactions for all of the tested putative W-linked transcripts, *AF18*, and *EF-1*α as well as RT-PCR reactions for the *doublesex* gene were initiated in a 25 μL reaction containing 1 μL template, 0.25 μmol/L of both the forward and reverse primers for each gene, 2.5 μL 2.5 mM dNTP, 5 μL PCR buffer with Mg^2+^ (Takara, Dalian) and 5 U Ex*Taq* DNA polymerase. The PCR or RT-PCR reactions were completed in an Eppendorf Mastercycler^®^ nexus (Eppendorf, Borsdorf, Leipzig, Germany) with a cycling program of denaturation at 95°C for 10 min, followed by 35 cycles of 95°C for 30 s, 60°C (62°C and 58°C for amplification of *AF18* with AF-18F and AF-18R) for 30 s and 72°C for 1 min, and a final extension of 72°C for 5 min. The resultant gPCR or RT-PCR products were separated at 200 V for about 15 min on 2% agarose gels in the 1 × TAE buffer, visualized by ethidium bromide staining, and documented using a ChampGel (Sagecreation, Beijing, China).

### Genome Walking

We used six gDNA samples extracted from three pairs of male and female pupae of *H. armigera* to clone the 5′-flanking sequences of *GUW1* and *AF18* by genome walking. As diagramed in Figure 5A, we digested 2.5 μg of each of the six gDNA samples with 2 μL of 5 U/μL *Msp*I (New England Biolab, Boston, MA, United States) in a 100 μL reaction in a 37°C water bath for 12 h. The digested gDNA samples were purified by TIANquick Midi Purification Kit (Tiangen Biotech). We then ligated 200 ng of each *Msp*I-digested DNA sample to the *Msp*I adapter at 16°C for 16 h in a 20 μL reaction containing 1 × T4 ligation buffer and 2 U T4 ligases (New England Biolab, Boston, MA, United States). The *Msp*I adapter was formed by annealing 1:1 ratio of the oligos *Msp*I*-a* and *Msp*I*-b* (Supplementary Table S2) by a 5-step descending heating program of 90, 70, 50, 30, and 16°C for 30 min, respectively.

We used the ligated products of the six gDNA samples as the templates to PCR-amplify the 5′-flanking sequences of *AF18* and *GUW1* with the general forward primer *Msp*I*-a* and the gene-specific reverse primers AF18-GSP and GUW1-118-R (Supplementary Table S2 and Figure 5A), respectively. The genome walking PCR reactions were carried out in a 25 μL reaction mixture containing 2.5 μL of PCR buffer, 0.5 μL of dNTP (10 mM), 0.25 μL of primestar GXL polymerase (5 U/mL), 0.5 μL of the specific primer pairs (20 mM), and 1 μL of the template DNA. The PCR cycling conditions consisted of denaturation at 95°C for 5 min, followed by five cycles of 95°C denaturation for 1min, annealing for 30 s from 65 to 60°C at a declining rate of 1°C/cycle, extension at 72°C for 1 min, 25 cycles of 95°C denaturation for 1 min, annealing at 60°C for 30 s, 72°C for 1 min, and a final extension at 72°C for 5 min. The PCR products were electrophoresed on a 2% agarose gel in the 1 × TAE buffer and visualized by ethidium bromide staining. The obtained bands of both *AF18* and *GUW1* were cloned into the pGEM^®^-T Easy Vector (Promega, Madison, WI, United States) and sequenced by Beijing Genomic Institute (BGI; Beijing, China).

### Data Analyses

The egg 454 sequence data were downloaded from NCBI SRA datasets (the accession number was SRP031603 and the run number was SRR1013685, SRR1015459, and SRR1015457) (Anantanawat, 2013). The downloaded SRA data were process into fastq format by the fastq-dump software, which is a part of SRA Toolkit. Then we mapped the egg transcriptome data to our unpublished *H. armigera* male pupa genome (including mitochondrion genome) using the NCBI local BLAST 2.2.26. The unmapped sequences were putatively derived from the W chromosome and 17 of them were selected for further verification.

*AF18* and *GUW1* as well as the 5′ flanking sequences obtained by genome walking were further analyzed by Blast to NCBI databases and *H. armigera* male genome to annotate them and verify their W chromosome uniqueness. Chi-square (χ^2^) test was used to determine if the sex ratio of *H. armigera* neonates determined by the *GUW1* marker is significantly different from the expected 1:1 sex ratio.

## Results

### Identification of W-Linked Candidate Transcripts

A total of 394,630 raw 454 sequence reads of *H. armigera* eggs (≤24 h post oviposition) of both sexes (Anantanawat, 2013) were downloaded from the NCBI website and then mapped to an unpublished male nuclear and mitochondrial genome of this species (Li et al., unpublished data). This transcriptome to genome blast revealed that 98.77% of the raw 454 sequence reads mapped at least one time to the male genomes. The remaining 4855 (1.23%) sequence reads failed to hit any scaffolds of the male genome and thus were putatively transcribed from the W chromosome of female eggs.

### Characterization of a Gene Unique to W Chromosome

In order to characterize one or more transcripts unique to W chromosome for development of PCR-based DNA markers for sexing male vs. female eggs, larvae, pupae, or even cells, we selected 24 non-transposon transcripts of >200 base pairs (bps) from the 4855 unmapped candidate sequences for designating a pair of PCR primers with Primer Premier 5.0 (Premier Biosoft International, Palo Alto, CA, United States). The software Primer Premier 5.0 found suitable primers (Supplementary Table S1) for 17 out of the 24 transcripts, but no suitable primers were found for the other 7 transcripts.

**FIGURE 1:**
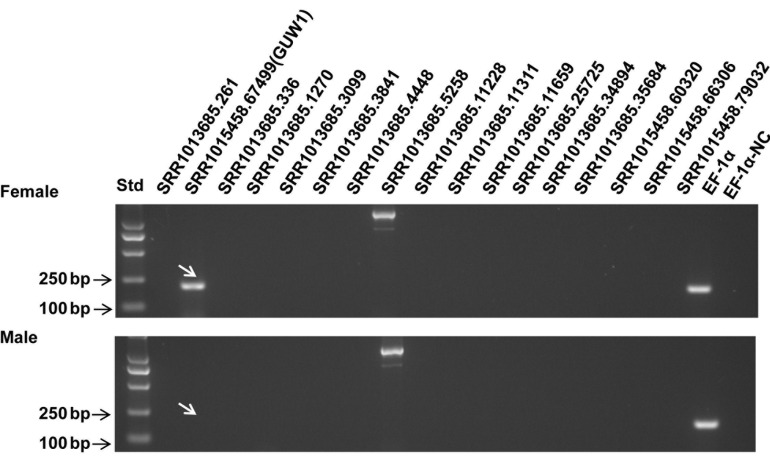
PCR detection of 17 putative W-linked sequences in a pair of *H. armigera* pupae. Genomic DNA extracted from one female **(top panel)** and one male **(bottom panel)** pupae of *H. armigera* were used as the templates to PCR-amplify *EF-1*α (as a positive control for autosome genes) and 17 selected putative W-linked sequences using the primers (Supplementary Table S1) designed based on their transcripts. The lane names starting with “SRR101” represent the PCR products of the 17 putative W-linked sequences. Std, molecular weight marker. EF-1α-NC, EF-1α negative control (no template control).

We prepared two gDNA samples from a pair of pupae sexed by the relative distance between their reproduction and excretion holes, and used the two gDNA samples as the templates to PCR-amplify the above 17 transcripts and *EF-1*α, a reference gene known to exist in both male and female individuals. As expected, *EF-1*α was detected in both the male and female pupae (Figure 1). No expected bands were detected for 15 out of the 17 transcripts in both the male and female pupae (Figure 1). One of the two remaining transcripts, SRR1017685.5288, like the reference gene *EF-1*α, was present in both the male and female pupae (Figure 1), and therefore is not a W-specific gene. By contrast, another remaining transcript, SRR1015458.67499, was detected only in the female pupa (Figure 1), and thus is probably a gene unique to W chromosome [here after named *gene unique to W 1* (*GUW1*)] and can be used to differentiate female vs. male cells and individuals at all developmental stages. Furthermore, Blast search of *GUW1* against the published male genome of the Australian population of *H. armigera* (Pearce et al., 2017) also failed to hit any scaffolds.

### Verification and Annotation of *GUW1* as a W-Specific Gene and of *AF18* as a Multicopy Transposon

**FIGURE 2:**
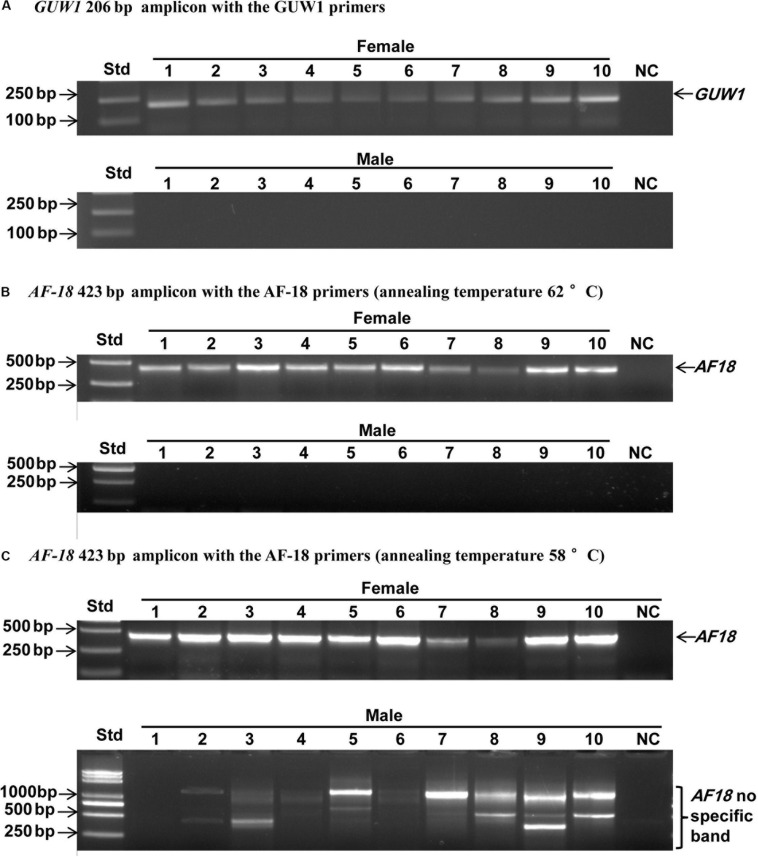
Sex identification of ten pairs of morphologically pre-sexed *H. armigera* pupae with the 206 bp *GUW1* amplicon **(A)** and the 423 bp *AF18* amplicon obtained at the annealing temperature of 62°C **(B)** and 58°C **(C)**, respectively. Genomic DNA samples were individually extracted from ten pairs of morphologically pre-sexed pupae and used as the templates to PCR-amplify *GUW1* with the primer pair GUW1-F and GUW1-R (Supplementary Table S2) and *AF18* with the primer pair AF18-F and AF18-R (Supplementary Table S2). Lanes numbered with 1–10 indicate the 10 female **(top panel)** or male **(bottom panel)** pupae in each figure. Std, molecular weight marker. NC, negative control (no template control) for *GUW1* **(A)** or *AF18* **(B–C)**.

**FIGURE 3:**
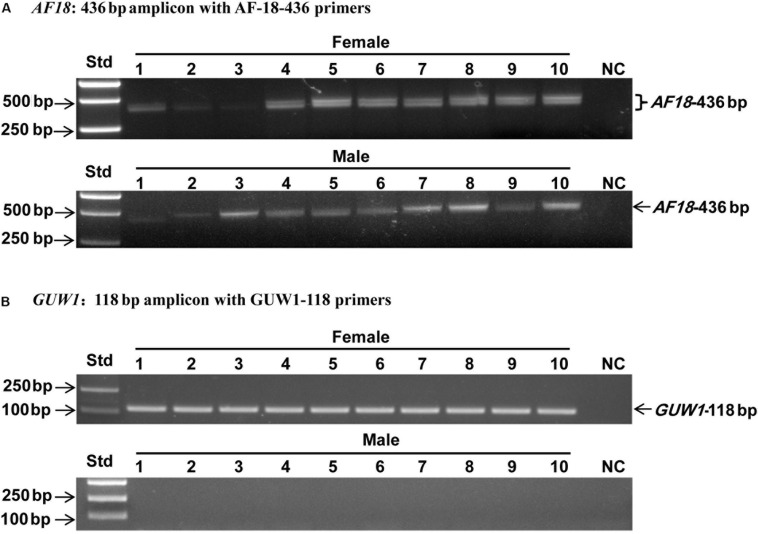
Sex identification of ten pairs of morphologically pre-sexed *H. armigera* pupae with the 436 bp *AF18* amplicon **(A)** and the 118 bp *GUW1* amplicon **(B)**. Genomic DNA samples from the same ten pairs of pupae as in Figure 2 were used as the templates to PCR-amplify *GUW1* with the primer pair GUW1-118-F and GUW1-118-R (Supplementary Table S2) and *AF18* with AF18-436-F3 and AF18-436-R3 (Supplementary Table S2). Lanes numbered with 1–10 indicate the 10 female (top panel) or male (bottom panel) pupae in each figure. Std, molecular weight marker. NC, negative control (no template control) for *AF18* **(A)** and *GUW1* **(B)**.

Three additional experiments were conducted to verify if *GUW1* and the previously identified female-specific DNA marker *AF18* (Niu et al., 2007) are W-specific sequences. First, we individually extracted gDNAs from 10 more pairs of morphologically pre-sexed pupae and tried to PCR-amplify both *GUW1* and *AF18* from the 10 pairs of pupae. Gel analysis of the PCR products showed the presence of *GUW1* (Figure 2A) and *AF18* (Figure 2B) in all 10 female pupae but not in all 10 male pupae. When the annealing temperature for PCR amplification of *AF18* decreased from 62°C (Figure 2B) to 58°C (Figure 2C), the expected *AF18* band occurred not only in the 10 female pupae but also in 4 (No. 2, 3, 5, and 9) out of the 10 male pupae (Figure 2C). Besides, 9 out of the 10 male pupae also had 1 or 2 obvious bands larger than the expected *AF18* band. When the annealing position of the reverse primer was moved only 13 bp downstream (compare the primers AF18-436-R3 and AF18-R in Supplementary Table S2), the expected *AF18* band appeared not only in the 10 female pupae but also in the 10 male pupae (Figure 3A). By contrast, shifting the forward primer 7 bp downstream (GUW1-118-F in Supplementary Table S2) and the reverse primer 81 bp upstream (GUW1-118-R in Supplementary Table S2) did not change the sex specificity of *GUW1* band in any of the 10 pairs of pupae (Figure 3B).

**FIGURE 4:**
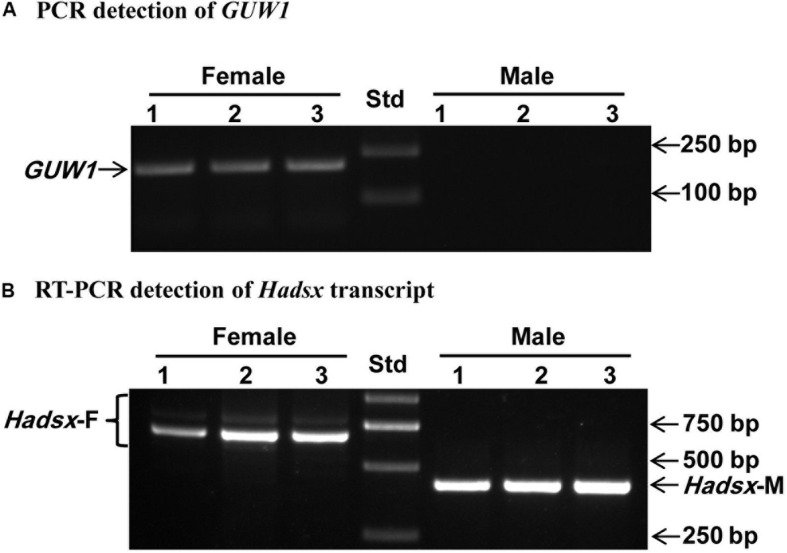
Sex identification of three pairs of morphologically pre-sexed *H. armigera* pupae with the 206 bp *GUW1* amplicon **(A)** and *Hadsx* transcript **(B)**. Genomic DNA and total RNA samples individually extracted from three pairs of male and female pupae were used as the templates to PCR-amplify *GUW1* with the primers GUW1-118-F and GUW1-118-R (Supplementary Table S2) and RT-PCR-amplify *Hadsx* transcripts with the primers Hadsx-F and Hadsx-R (Supplementary Table S2), respectively. Lanes numbered with 1–3 indicate the three female (top panel) or male (bottom panel) pupae in each figure. Std, molecular weight marker.

Second, we used the gDNA and RNA samples from 3 pairs of pupae as the templates to amplify *GUW1* and the sex-unique transcript isoforms of *Hadsx* gene, respectively. According to Wang et al. (2014), the primer pairs we used for *Hadsx* (Hadsx-F and Hadsx-R in Supplementary Table S2) should yield four bands of 668/683 bp (not separable on gels) and 797/812 bp (not separable on gels) in females, but one band of 419 bp in males. Gel analysis of the *GUW1* and *Hadsx* PCR or RT-PCR products showed that all the 3 pupae with the female-unique transcript isoforms of *Hadsx* had the expected *GUW1* band, whereas those with the male-unique transcript isoform of *Hadsx* lacked the *GUW1* band (Figure 4).

**FIGURE 5:**
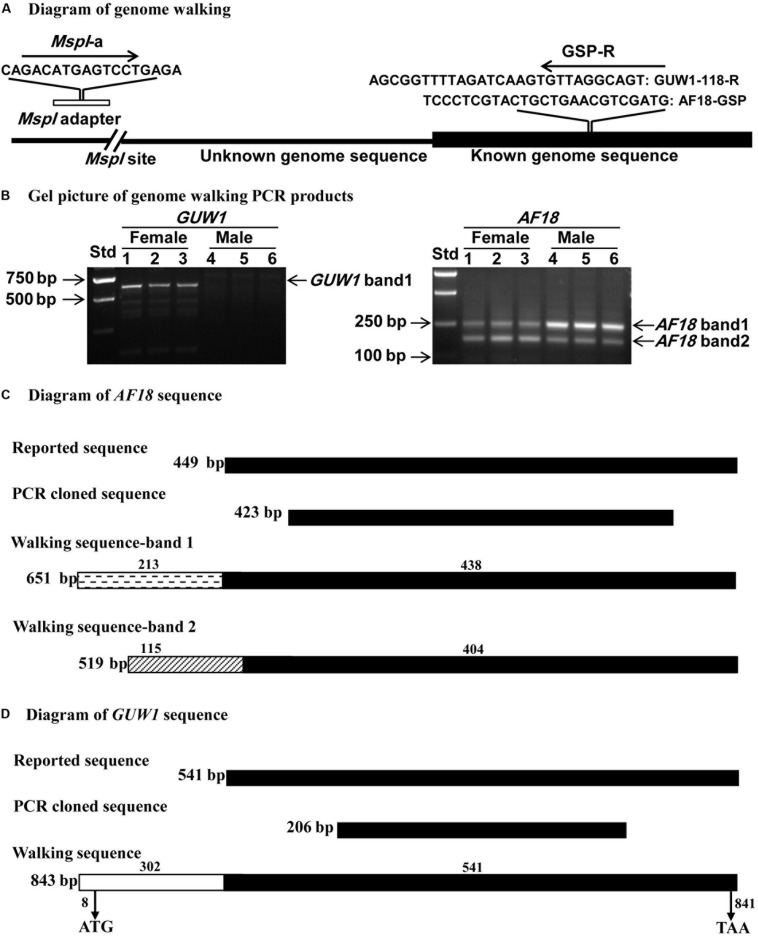
Schematic alignments of *GUW1* and *AF18* with their 5′ flanking sequence obtained by genome walking. Genome DNA samples from the same three pairs of male and female pupae as in Figure 4A were completely digested by *Msp*I and ligated to *Msp*I adaptor. The ligation mixtures were used as the templates to PCR-amplify the 5′ unknown flanking sequences of *GUW1* and *AF18* using the general primer *Msp*I-a and the gene-specific primers GUW1-118-R and AF18-GSP (Supplementary Table S2), respectively **(A)**. The obtained genome walking PCR products of *GUW1* (left panel in **B**) and *AF18* (right panel in **B**) from the three pairs of pupae were separated on 2% agarose gels **(B)**. The two genome walking bands of *AF18* from each of the six pupae and the single walking band of *GUW1* from the three female pupae were sequenced and schematically aligned with the reported and cloned *AF18* **(C)** and *GUW1* sequences **(D)**, respectively. Filled black boxes represent the known (reported) sequences of *AF18* or *GUW1*, whereas clear (*GUW1*), cross-striped (*AF18* band 1) or dashed (*AF18* band 2) boxes represent the 5′ flanking sequences of the two markers identified by our genome walking experiment. The lengths of the 5′ flanking and reported (known) parts of the three walking sequence are placed above each box. The total length of each sequence is shown on the right side of each box. The positions of start and stop codons of the *GUW1* open reading frame are shown below the walking sequence box. Std, molecular weight marker.

We also conducted genome walking to amplify the 5′ flanking sequences of *AF-18* and *GUW1* from the above three pairs of pupae (Figure 5A). Gel analysis of the genome walking products revealed one band of 635 bp for *GUW1* only in the three female pupae, but two bands of 158 and 290 bp for *AF18* in both the three female and three male pupae (Figure 5B). Sequencing of the two *AF18* bands reveals that they not only miss the first 11 (band 1) and 45 bp (band 2) of the reported *AF18* sequence respectively, but also share no significant similarity in their 5′ flanking sequences (Figure 5C and Supplementary Figure S1). These results indicate that the two bands represent two different copies of *AF18* located on Z chromosome or autosome, whereas the previously reported female-specific copy (Niu et al., 2007) locates on W chromosome. Careful examination of the reported 449 bp *AF18* sequence (Niu et al., 2007; Figure 5C) confirms that it is a MITE (Miniature inverted repeat transposable element; named *MITE1_Har*) because it has an AT-rich internal sequence (65% = 35%A + 30%T), a pair of 8-bp terminal inverted repeats (TIRs, left TIR = GTGTCCCT), and lacks coding potential. Blast search with *MITE1-Har* as the query yielded 137 significant hits (e-value ≤ 1.0e-10) to the *H. armigera* male genome scaffolds (Supplementary Table S3), indicating this transposon has multiple copies present on both Z chromosome and autosomes. We listed 10 representative copies of *MITE1_Har* in Table 1.

**TABLE 1:** Ten representative copies of *MITE1_Har*.

Name	Location	length	TIR	TSD
*HaMITE1* (i.e., *AF18*)	W chromosome	449	GTGTCCCT	*
*HaMITE1-1*	Scaffold235	2413	–	TCC
*HaMITE1-2*	Scaffold710	2412	–	ATTTTT
*HaMITE1-3*	Scaffold570	301	TTTTGA	TTTGA
*HaMITE1-4*	Scaffold169	2412	–	ATTTTT
*HaMITE1-5*	Scaffold57	2412	–	ATTTTT
*HaMITE1-6*	Scaffold441	300	TTTTGA	TTTGA
*HaMITE1-7*	Scaffold877	300	TTTTGA	TTTGA
*HaMITE1-8*	Scaffold1156	285	–	GCCT
*HaMITE1-9*	Scaffold293	106	AAGAAA	AAT
*HaMITE1-10*	Scaffold66	46	GAG	TTTAG

*Cannot locate it on the W chromosome. –No significate TIR element.

**FIGURE 6:**
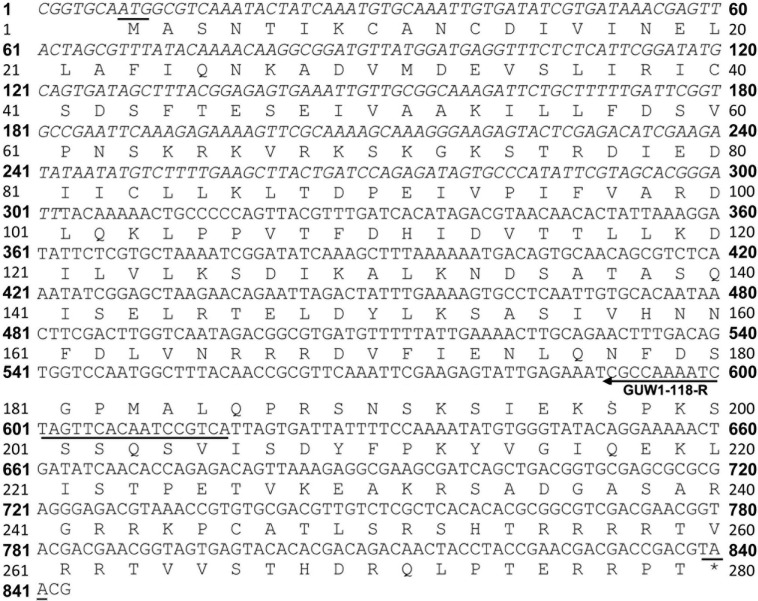
Nucleotide and deduced amino acid sequence of *GUW1*. The merged sequence of the reported *GUW1* transcript (upright) and its 5′ flanking sequence (italicized) has an open reading frame (ORF) encoding a protein of 277 amino acids. The start and stop codons of the ORF are underlined. The annealing direction and position of the gene-specific primer GUW1-118R (Supplementary Table S2) used for genome walking of *GUW1* are depicted with an arrowed line.

In contrast, sequencing of the only genome walking band for *GUW1* from female pupae shows no 5′ end missing of the reported transcript (SRR1015458.67499) sequence (Figures 5D, 6), suggesting that the band is originated from the same locus with SRR1015458.67499 on W chromosome. Consistent with the absence of a genome walking band for *GUW1* in the male pupae, BLASTn search with the assembled/edited sequence of the reported transcript plus the genome walking band (Figure 6) as the query produced no significant hits to the male *H. armigera* genome scaffolds. The assembled/edited *GUW1* sequence has an open reading frame encoding a protein of 277 amino acids (aa) (Figure 6). BLASTn search with *GUW1* as the query against the NCBI nucleotide collection found no similar sequences. BLASTp search against the NCBI protein database showed that the predicted GUW1 protein shares the highest amino acid sequence identity (69%) with the hypothetical protein RR48_05862 (GenBank accession# KPJ14768, 446 aa) predicted from *Papilio machaon* male adult genome (Li et al., 2015) in their first N-terminal 234 amino acids (query coverage 84%, e-value = 3e-10^8^). BLASTn search against the publicly available chromosome-level genomes of eight lepidopteran species detected five partial alignments (in the 5′ 683 bp) with an identity of =0.74% and a coverage of =0.81% (2 on autosome 7, 2 on autosome 15, and 1 on autosome 27) in the genome of *Spodoptera litura*, 10 short partial alignments (in the 5′ 376 bp) with an identity of =0.73% and a coverage of =0.44% (4 on W chromosome, 1 on autosome 14, 1 on autosome 16, 1 on autosome 5, 1 on autosome 7, 1 on autosome 18 and 1 on autosome 19) in the genome of *Trichoplusia ni*, and no significant hits in the genomes of *C. pomonella* (female), *Spodoptera frugiperda* (female), *Ephestia kuehniella* (W chromosome only), *Dendrolimus punctatus* (female), *Zerene cesonia* (male), and *Danaus plexippus* (female) (Table 2). tBLASTn search against the genomes of the 8 lepidopteran species revealed multiple 5′ partial alignments (in the N-terminal 225 aa) with a coverage of =0.80% and an identity of =65%. in the genomes of *S. litura*, *T. ni*, *E. kuehniella, Z. cesonia*, and *D. plexipus* but no hits in the other three species (Table 2).

**TABLE 2:** Significant GUW1 hits found in the chromosome-level genomes of eight lepidopteran species.

Species	BLASTn hits (Score/Cover%/E value/Identity%)^b^	tBLASTn hits (Score/Cover%/E value/Identity%)^b^	References
*Cydia pomonella* (♀)	–	–	Wan et al., 2019
*Spodoptera litura* (♂)	Chr7(516/81/2e-121/74.42)	Chr27(969/80/8e-89/64.89)	Cheng et al., 2017
	Chr15(478/80/6e-121/74.37)	Chr5(730/80/1e-88/65.02)	
	Chr27(439/81/2e-121/74.42)	Chr7(1218/80/4e-88/64.44)	
*Spodoptera frugiperda* (♀)	–	–	Xiao et al., 2020
*Trichoplusia ni* (♀)	W(792/44/1e-53/72.61)	W(1817/80/2e-68/55.11)	Fu et al., 2018;
	Chr14(158/16/2e-13/76.92)	Chr16(933/65/1e-44/54.43)	Chen et al., 2019
	Chr16(157/19/2e-13/76.92)	Chr14(696/64/1e-44/54.43)	
*Ephestia kuehniella* (W chr)	–	S6870(76/29/1e-20/61.84)	Traut et al., 2013
		S7568 (73/28/7e-20/61.64)	
		S3732 (63/47/6e-18/47.31)	
*Dendrolimus punctatus*(♀)	–	–	Zhang et al., 2020
*Zerene cesonia* (♂)	–	Z(156/66/4e-42/45.21)	Luis et al., 2020
		ChrXIX(127/61/5e-32/38.29)	
		ChrXII(122/49/3e-30/45.65)	
*Danaus plexippus* (♀)	–	Chr7(127/47/7e-32/51.85)	Mongue et al., 2017
		Chr18(91/40/3e-19/45.13)	
		Chr10(69/48/6e-12/34.23)	

*^a^Only the lepidopteran species with a chromosome-level genome or W chromosome genome assembly are listed here. ♂ indicates male genome, ♂ indicates female genome, W chr indicates W chromosome only. ^b^Only the top three hits are presented for each species. – means no significant hits.*

### Application of *GUW1*-Based PCR Gel Analysis to Sex Young *H. armigera* Larvae and Cell Line

**FIGURE 7:**
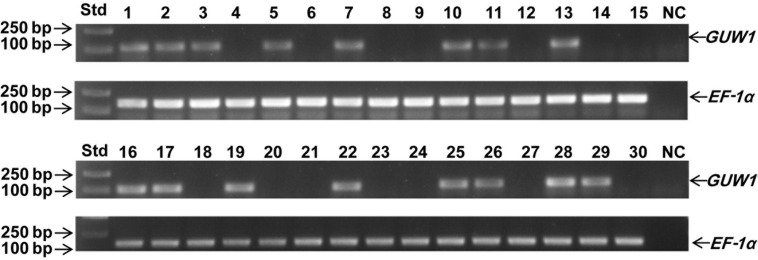
Test application of the 118 bp *GUW1* amplicon for sex identification of 30 neonates of *H. armigera*. Genomic DNA samples individually extracted from 30 neonates of <5 h were used as the templates to PCR-amplify the W-linked *GUW1* with the primer pair GUW1-118-F and GUW1-118-R (Supplementary Table S2) and the autosomal gene *EF-1*α with the primer pair EF1α-F and EF1α-R (Supplementary Table S2). Lanes numbered with 1–30 represent the 30 neonates. Std, molecular weight marker. NC, negative control (no template control) for *GUW1* and *EF-1*α.

To test the utility of the W-specific *GUW1* gene for sexing young larvae, we randomly selected 30 neonates of *H. armigera* and extracted gDNA from each of the 30 larvae. PCR gel analysis with the gene-specific primers for the W-specific marker gene *GUW1* (GUW1-118-F and GUW1-118-R in Supplementary Table S2) and the autosomal gene *EF-1*α (EF1α-F and EF1α-R in Supplementary Table S2) showed the presence of the *EF-1*α band in all the 30 first-instar larvae (Figure 7). By contrast, the *GUW1* band was detected only in 16 out of the 30 neonates. Chi-square (χ^2^) test confirmed that the sex ratio (16 female: 14 male = 1.14:1.0) obtained with the *GUW1* marker was not significantly different from the expected 1:1 sex ratio (χ^2^ value = 0.067, *P* = 0.80).

**FIGURE 8:**
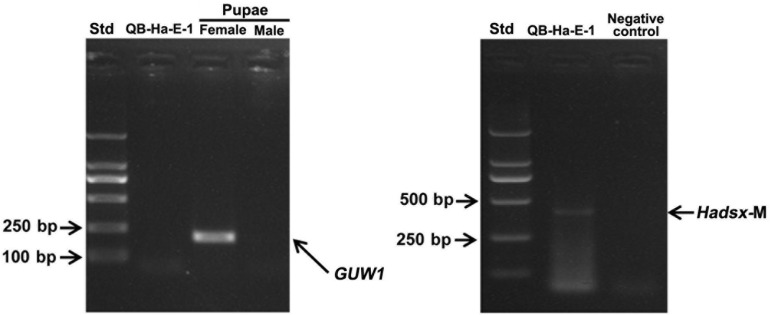
Sex identification of *H. armigera* cell line QB-Ha-E-1 with the 206 bp *GUW1* amplicon **(left panel)** and *Hadsx* transcript **(right panel)**. The gDNA samples extracted from a pair of pre-sexed pupae as well as the gDNA and total RNA samples from the *H. armigera* cell line QB-Ha-E-1 were used as the templates to PCR-amplify *GUW1* **(left panel)** with the primer pair GUW1-F and GUW1-R (Supplementary Table S2) and RT-PCR-amplify *Hadsx* transcripts **(right panel)** with the primers Hadsx-F and Hadsx-R (Supplementary Table S2), respectively. Std, molecular weight marker.

To test the utility of the W-specific *GUW1* gene for sexing *H. armigera* cell lines, we extracted gDNA and total RNA from the embryo cell line QB-Ha-E-1 of *H. armigera* (Zheng et al., 2010) and gDNA from a pair of female and male pupae. We then used the obtained gDNA and RNA samples as the templates to PCR-amplify *GUW1* and RT-PCR-amplify *Hadsx* transcripts, respectively. Gel analysis of the gDNA PCR products detected the *GUW1* band in the female pupa, but not in the embryo cell line QB-Ha-E-1 and male pupa (see the left panel in Figure 8). Consistent with the absence of *GUW1* gene in QB-Ha-E-1, gel analysis of the RT-PCR products showed that QB-Ha-E-1 had the male-unique transcript isoform of *Hadsx* (see the right panel in Figure 8).

## Discussion

The absence of conspicuous sexual dimorphism prior to pupation makes it necessary to identify W chromosome-linked genes or sequences for accurate and reliable determination of the gender of individual eggs and larvae of *H. armigera.* Recent studies have demonstrated that characterization of Y or W chromosome-linked sequences or genes can be achieved through segregation and linkage analysis of transcriptomic alleles from parents to their F_1_ male and female progenies (Bergero and Charlesworth, 2011; Chibalina and Filatov, 2011), comparison of male vs. female transcriptomes of an inbred line (Muyle et al., 2012), and contrast of male vs. female genomes of a given species (Portela et al., 2010; Chen et al., 2012; Koerich et al., 2016; Fraïsse et al., 2017). Unlike the above three omics approaches that rely on either transcriptome-transcriptome contrast (Bergero and Charlesworth, 2011; Chibalina and Filatov, 2011; Muyle et al., 2012) or genome-genome subtraction (Portela et al., 2010; Chen et al., 2012; Koerich et al., 2016; Fraïsse et al., 2017), here we identified 4855 putative W-linked transcript sequences of *H. armigera* by mapping an egg transcriptome of both sexes (Anantanawat, 2013) to a male genome of this species (Li et al., unpublished data). This approach, like the other three omics approaches, is also based on the fact that sequences unique to Y or W chromosome are not present in the genome of a female or male.

Genomic PCR analysis of a small set of 17 non-transposon transcripts selected from the 4855 putative W-linked transcript sequences found only 1 W-linked sequence (i.e., the true positive *GUW1*; detected only in the female pupa) but 15 negatives (not detected in pupae of both sexes) and 1 false positive (detected in pupae of both sexes) (Figure 1). Such a high ratio (15/17) of negatives is probably caused largely by the repeat-rich introns commonly present in the sex-specific chromosome-linked genes (Gatti and Pimpinelli, 1983; Carvalho et al., 2000; Reugels et al., 2000). These giant introns may locate within one primer or between the primer pairs of the 15 negatives, leading to the failure of the corresponding PCR reactions. Alternatively, some of the 15 negative sequences could be derived from the microorganisms (symbionts or parasites) that lived only in the eggs used for generation of the egg transcriptome (Anantanawat, 2013) and thus were not detected in both male and female pupae. Lastly, inter-population differences of *H. armigera* could be the third possible reason, whereby some of the 15 negative sequences present in the *Bt* resistant Australian population employed for production of the egg transcriptome (Anantanawat, 2013) might be deleted in the Chinese population used in this study.

Several additional experiments and analyses were conducted to verify the W chromosome uniqueness and identities of the only positive *GUW1* identified here and the previously-reported female-specific RAPD marker *AF18* (Niu et al., 2007). Detection of the *AF18* band in both male and female pupae at a lower annealing temperature (from 62 to 58°C; Figure 2C) or at a different annealing position for the reverse primer (13 bp downstream; Figure 3A), observation of two genome walking bands in both male and female pupae (Figure 5B), and lack of sequence similarity between the 5′ flanking sequences of the two genome walking bands and the 5′-end of the reported *AF18* sequence (Niu et al., 2007; Figure 5C) strongly suggest that *AF18* has multiple copies in the genome and some of its copies also sit on Z chromosome and/or autosomes. Consistent with this inference, our sequence analyses confirm that *AF18* sequence is a MITE (named *MITE1_Har*) with 137 significant hits (e-value ≤ 1.0e-10) to the *H. armigera* male genome scaffolds (Table 1 and Supplementary Table S3). This is not unexpected as the lepidopteran W chromosomes are rich in transposons (Abe et al., 2005; Fuková et al., 2007; Traut et al., 2013).

In contrast, multiple evidence including detection of *GUW1* only in the female pupae regardless of the annealing positions of the two primers (Figures 2A, 3B), coupling of *GUW1* with female-unique splicing of *Hadsx* transcript (Figure 4), appearance of a single genome walking band only in the female pupae (Figures 5B,D), reliable sexing of a “tester set” of 30 neonates with *GUW1* (Figure 7) and one *H. armigera* cell line (Figure 8), and its lack of no significant hits to the male genomes of both the Chinese (Li et al., unpublished data) and Australian (Pearce et al., 2017) populations confirm that *GUW1* is indeed a single copy sequence unique to W chromosome. Translation analysis reveals that GUW1 encodes a protein of 277 amino acids (Figure 6), whose first N-terminal 234 amino acids has multiple tBLASTn hits of 34-65% similarity on several autosomes of *S. litura*, *D. plexippus*, *T. ni*, and *Z. cesonia*, Z chromosome of *Z. cesonia*, and W chromosome of *T. ni* and *E. kuehniella* (Table 2), and is 69% identical to the N-terminal half (446 amino acids in total) of the hypothetical protein RR48_05862 of *P. machaon*. However, none of the tBLASTn hits on the W chromosome of *T. ni* and *E. kuehniella* represents an intact protein-coding gene (Traut et al., 2013; Fu et al., 2018; Chen et al., 2019). And RR48_05862 is not a W chromosome-unique protein-coding gene since it was annotated from the male adult genome of *P. machaon* (Li et al., 2015). Further experiments are required to decipher the functions and evolutionary relationship between *GUW1* and RR48_05862.

The W chromosome in Lepidoptera is replete with transposons but poor in protein-coding genes (Sahara et al., 2003, 2012; Abe et al., 2005; Fuková et al., 2007; Traut et al., 2013). Comprehensive gene-based surveys have not found a single protein-coding gene on the W chromosome in *Heliconius melpomene* (Pringle et al., 2007), *Bicyclus anynana* (Beldade et al., 2009), *Plutella xylostella* (Baxter et al., 2011), *C. pomonella* (Wan et al., 2019), *S. litura* (Cheng et al., 2017), *S. frugiperda* (Xiao et al., 2020), and *T. ni* (Fu et al., 2018; Chen et al., 2019). High-throughput sequencing of the W chromosome of the flour moth (*E. kuehniella)* has not detected a single protein-coding gene either (Traut et al., 2013). Even in the most extensively-studied lepidopteran *B. mori*, no protein-coding gene but a piRNA gene (Fem) has been identified recently on the W chromosome (Kiuchi et al., 2014; Fujii et al., 2015; Zhang et al., 2018). The prediction of 14 protein-coding loci on the putative W-linked scaffolds of *D. plexippus* (Mongue et al., 2017) represents one exception, but the W chromosome of this species is probably a neo-W chromosome—fusion of ancient W with an autosome, rather than a typical fully-degenerated W chromosome (Mongue et al., 2017). The other two exceptions found so far are a truncated W homolog of the Z-linked *period* gene in *Antheraea pernyi* (Gotter et al., 1999) and a W homolog of the Z-linked *laminin A* gene in the peppered moth (*Biston betularia*; Van’t Hof et al., 2013). These two W homologs might have resulted from the Z-linked *period* or *laminin A* gene by transposon-mediated ectopic recombination between the Z and W chromosomes (Van’t Hof et al., 2013). By contrast, *GUW1* characterized and verified at the transcription level in this study is a W-specific protein coding gene and is not present on the Z or autosome in *H. armigera*. Thus, *GUW1* is the first W-specific protein coding gene found in Lepidoptera and represents a breakthrough that provides new insights into the evolution of the W chromosome in butterflies and moths.

## Data Availability Statement

All datasets generated for this study are included in the article/Supplementary Material.

## Author Contributions

XL and ZD conceived and designed the experiments, and analyzed the data. ZD and YZ performed the experiments. XL, ZD, MZ, YZ, XN, CL, and JH wrote the manuscript. All authors have read and approved the manuscript for publication.

## Disclaimer

Mention of trade names or commercial products in this article is solely for the purpose of providing specific information and does not imply recommendation or endorsement by the U.S. Department of Agriculture.

## Conflict of Interest

The authors declare that the research was conducted in the absence of any commercial or financial relationships that could be construed as a potential conflict of interest.
