# Supplementary material for: Characterization of the First W-Specific Protein-Coding Gene for Sex Identification in Helicoverpa armigera
Source: Front Genet. 2020 Jun 19;11:649. doi: 10.3389/fgene.2020.00649 (PMC7317607; doi:10.3389/fgene.2020.00649)
Supplement: Supplementary file 1 [file Data_Sheet_1.docx]

**Table S1. The primers designed for amplification of the 17 selected putative W-linked transcripts**

| Primer name | Primer sequence (5’ to 3’) | Length | Product Size |
| --- | --- | --- | --- |
| SRR1013685.261F | AACCGAAAACGTACCTAAACCT | 22 | 233 |
| SRR1013685.261R | CCCGTAACGTTTAACTTTCGT | 21 |  |
| SRR1013685.336F | AGCTGCTCGTCTGAACACAG | 20 | 201 |
| SRR1013685.336R | GTACGTTGACAAACGACGATC | 21 |  |
| SRR1013685.1270F | TCGACGTGCGAATTTTTAAT | 20 | 214 |
| SRR1013685.1270R | GGGATAGAATGAGGATTTGCA | 21 |  |
| SRR1013685.3099F | TAACCGAAAACGGTACCAAA | 20 | 205 |
| SRR1013685.3099R | CGTTACGGTTAAACGTTTCG | 20 |  |
| SRR1013685.3841F | TCGTCGTAGGGTACGTACGT | 20 | 210 |
| SRR1013685.3841R | CGTTACCGTAACCGTTAACG | 20 |  |
| SRR1013685.4448F | CTAGACTCGACGTGCGTCAT | 20 | 212 |
| SRR1013685.4448R | TCTTGGCTGTGTGACAGAGG | 20 |  |
| SRR1013685.5258F | ACGGTCGTCTTCTACCGTTAC | 21 | 203 |
| SRR1013685.5258R | GTTACGGTACGACGAACGAC | 20 |  |
| SRR1013685.11228F | GGCATTGCTTATCACAGAGG | 20 | 205 |
| SRR1013685.11228R | CGGAGAGAACGAGTACTCCA | 20 |  |
| SRR1013685.11311F | ACGGTTTTACGGTTTTACGG | 20 | 202 |
| SRR1013685.11311R | CGTTACGTACCGTACCGGTA | 20 |  |
| SRR1013685.11659F | CTCGACGTGCTTTATTATTTGAC | 23 | 214 |
| SRR1013685.11659R | AGGACCTATGTGGTCAGCAA | 20 |  |
| SRR1013685.25725F | GACACAACGACACGAGCATA | 20 | 245 |
| SRR1013685.25725R | CCGTTAAACCGTAACGTACG | 20 |  |
| SRR1013685.34894F | CGGTAACGGTAACGACGTAC | 20 | 203 |
| SRR1013685.34894R | TCCGTACGACGTTACGACTT | 20 |  |
| SRR1013685.35684F | GCCGACGTACTAACGTAACG | 20 | 200 |
| SRR1013685.35684R | GGTTTACGGGTTACGGGTTA | 20 |  |
| SRR1015458.60320F | CGACGTAACGGACGTACAAT | 20 | 220 |
| SRR1015458.60320R | TTGTCTACCGGGTCTTACGTAG | 22 |  |
| SRR1015458.66306F | CGTAGGGTTCCTAGGGGTTA | 20 | 212 |
| SRR1015458.66306R | CCGGTTAAATCCCGTTAAAC | 20 |  |
| SRR1015458.67499F | CAATAGACGGCGTGATGTTT | 20 | 214 |
| SRR1015458.67499R | ATCGCTTCGCCTCTTTAACT | 20 |  |
| SRR1015458.79032F | ACCCAAGACGGAACCTTAAC | 20 | 200 |
| SRR1015458.79032R | TCGTCTAGTTGGGTCCCTAAA | 21 |  |

**Table S2. The primer used for verification the W uniqueness of *AF18* and *GUW1***

| Primer Name | Primer Sequence | Anneal Temp(°C) | | Products Size(bp) |
| --- | --- | --- | --- | --- |
| GUW1-F  GUW1-R | CAATAGACGGCGTGATGTTT  ATCGCTTCGCCTCTTTAACT | | 60 | 206 |
| AF18-F  AF18-R | CGAACACCGACGCTACCGCAAG  TGCAGGCCCCATTCAGCTAGTCGT | | 62/60/58 | 423 |
| AF18-436-F3 AF18-436-R3 | CGAACACCGACGCTACCGCAAGCAATAC  GTCCCTGTACAAATGCAGGCCCCATTCA | | 60 | 436 |
| GUW1-118-F GUW1-118-R^a^ | CGGCGTGATGTTTTTATTGAAAACTTGC  TGACGGATTGTGAACTAGATTTTGGCGA | | 60 | 118 |
| EF1α-F  EF1α-R | GAAGTCAAGTCCGTGGAGATG  GACCTGTGCTGTGAAGTCG | | 60 | 170 |
| AF18-GSP | GTAGCTGCAAGTCGTCATGCTCCCT | | - |  |
| *MspI-a*  *MspI-b* | CAGACATGAGTCCTGAGA  5′ (p)-CGTCTCAGGACTCAT-NH2-3′ | | 55 |  |
| Hadsx-F Hadsx-R | GGTGCTCGTGATCCTCAACTATG  CTTAACAGATCTAGCACTAGGTGTCCTG | | 60 | 419 in males; 668/683 and 797/812 in females |

^a^ Also used as the gene-specific primer for genome walking of *GUW1.*

**Table S3 The 137 male *H. armigera* genome scaffolds hit by *AF18***

| Sequences producing significant alignments | Score(bits) | E Value |
| --- | --- | --- |
| scaffold877 | 898 | 0 |
| scaffold809 | 884 | 0 |
| scaffold235 | 866 | 0 |
| scaffold534 | 850 | 0 |
| scaffold710 | 842 | 0 |
| scaffold169 | 842 | 0 |
| scaffold57 | 842 | 0 |
| scaffold10 | 842 | 0 |
| scaffold473 | 839 | 0 |
| scaffold570 | 835 | 0 |
| scaffold441 | 827 | 0 |
| scaffold49 | 827 | 0 |
| scaffold424 | 819 | 0 |
| scaffold233 | 819 | 0 |
| scaffold200 | 819 | 0 |
| scaffold192 | 819 | 0 |
| scaffold73 | 819 | 0 |
| scaffold21 | 819 | 0 |
| scaffold278 | 813 | 0 |
| scaffold6 | 813 | 0 |
| scaffold666 | 811 | 0 |
| scaffold499 | 811 | 0 |
| scaffold56 | 811 | 0 |
| scaffold368 | 799 | 0 |
| scaffold698 | 753 | 0 |
| scaffold1023 | 745 | 0 |
| scaffold242 | 737 | 0 |
| scaffold486 | 696 | 0 |
| scaffold343 | 684 | 0 |
| scaffold41 | 658 | 0 |
| scaffold521 | 654 | 0 |
| scaffold481 | 654 | 0 |
| scaffold468 | 654 | 0 |
| scaffold82 | 654 | 0 |
| scaffold11 | 654 | 0 |
| scaffold126 | 640 | 0 |
| scaffold1156 | 636 | 0 |
| scaffold643 | 636 | 0 |
| scaffold437 | 636 | 0 |
| scaffold327 | 636 | 0 |
| scaffold283 | 636 | 0 |
| scaffold248 | 636 | 0 |
| scaffold155 | 636 | 0 |
| scaffold1056 | 628 | e-178 |
| scaffold43 | 628 | e-178 |
| scaffold150 | 620 | e-176 |
| scaffold177 | 605 | e-171 |
| scaffold4 | 543 | e-153 |
| scaffold400 | 523 | e-147 |
| scaffold196 | 482 | e-134 |
| scaffold78 | 274 | 8.00E-72 |
| scaffold66 | 268 | 5.00E-70 |
| scaffold491 | 248 | 5.00E-64 |
| scaffold383 | 248 | 5.00E-64 |
| scaffold293 | 248 | 5.00E-64 |
| scaffold107 | 248 | 5.00E-64 |
| scaffold45 | 248 | 5.00E-64 |
| scaffold44 | 248 | 5.00E-64 |
| scaffold18 | 248 | 5.00E-64 |
| scaffold1068 | 246 | 2.00E-63 |
| scaffold448 | 246 | 2.00E-63 |
| scaffold447 | 246 | 2.00E-63 |
| scaffold434 | 246 | 2.00E-63 |
| scaffold257 | 246 | 2.00E-63 |
| scaffold201 | 246 | 2.00E-63 |
| scaffold62 | 246 | 2.00E-63 |
| scaffold654 | 240 | 1.00E-61 |
| scaffold231 | 240 | 1.00E-61 |
| scaffold191 | 240 | 1.00E-61 |
| scaffold58 | 240 | 1.00E-61 |
| scaffold19 | 238 | 5.00E-61 |
| scaffold626 | 236 | 2.00E-60 |
| scaffold442 | 236 | 2.00E-60 |
| scaffold35 | 236 | 2.00E-60 |
| scaffold1 | 236 | 2.00E-60 |
| scaffold16 | 220 | 1.00E-55 |
| scaffold871 | 202 | 2.00E-50 |
| scaffold93 | 192 | 2.00E-47 |
| scaffold309 | 184 | 6.00E-45 |
| scaffold1124 | 167 | 1.00E-39 |
| scaffold869 | 167 | 1.00E-39 |
| scaffold799 | 167 | 1.00E-39 |
| scaffold795 | 167 | 1.00E-39 |
| scaffold730 | 167 | 1.00E-39 |
| scaffold628 | 167 | 1.00E-39 |
| scaffold627 | 167 | 1.00E-39 |
| scaffold557 | 167 | 1.00E-39 |
| scaffold546 | 167 | 1.00E-39 |
| scaffold500 | 167 | 1.00E-39 |
| scaffold410 | 167 | 1.00E-39 |
| scaffold391 | 167 | 1.00E-39 |
| scaffold384 | 167 | 1.00E-39 |
| scaffold371 | 167 | 1.00E-39 |
| scaffold350 | 167 | 1.00E-39 |
| scaffold286 | 167 | 1.00E-39 |
| scaffold243 | 167 | 1.00E-39 |
| scaffold187 | 167 | 1.00E-39 |
| scaffold149 | 167 | 1.00E-39 |
| scaffold142 | 167 | 1.00E-39 |
| scaffold141 | 167 | 1.00E-39 |
| scaffold136 | 167 | 1.00E-39 |
| scaffold129 | 167 | 1.00E-39 |
| scaffold125 | 167 | 1.00E-39 |
| scaffold120 | 167 | 1.00E-39 |
| scaffold111 | 167 | 1.00E-39 |
| scaffold81 | 167 | 1.00E-39 |
| scaffold75 | 167 | 1.00E-39 |
| scaffold72 | 167 | 1.00E-39 |
| scaffold59 | 167 | 1.00E-39 |
| scaffold30 | 167 | 1.00E-39 |
| scaffold3 | 167 | 1.00E-39 |
| scaffold99 | 165 | 5.00E-39 |
| scaffold320 | 159 | 3.00E-37 |
| scaffold229 | 159 | 3.00E-37 |
| scaffold110 | 159 | 3.00E-37 |
| scaffold1273 | 155 | 5.00E-36 |
| scaffold621 | 155 | 5.00E-36 |
| scaffold580 | 155 | 5.00E-36 |
| scaffold399 | 155 | 5.00E-36 |
| scaffold79 | 155 | 5.00E-36 |
| scaffold199 | 153 | 2.00E-35 |
| scaffold1003 | 151 | 8.00E-35 |
| scaffold77 | 151 | 8.00E-35 |
| scaffold159 | 149 | 3.00E-34 |
| scaffold33 | 149 | 3.00E-34 |
| scaffold646 | 145 | 5.00E-33 |
| scaffold116 | 145 | 5.00E-33 |
| scaffold503 | 139 | 3.00E-31 |
| scaffold13 | 139 | 3.00E-31 |
| scaffold311 | 135 | 5.00E-30 |
| scaffold466 | 123 | 2.00E-26 |
| scaffold664 | 119 | 3.00E-25 |
| scaffold433 | 119 | 3.00E-25 |
| scaffold583 | 117 | 1.00E-24 |
| scaffold64 | 100 | 3.00E-19 |
| scaffold1466 | 84 | 2.00E-14 |
| scaffold148 | 84 | 2.00E-14 |

**Figure S1. Alignment of AF18 original sequence with the sequences of its two genome walking bands.** The annealing direction and position of the gene-specific primer AF18-GSP (Table S3) used for genome walking of *AF18* are depicted with an arrowed line and the primer name.
